# Stimuli-Responsive DNA Hydrogel Design Strategies for Biomedical Applications

**DOI:** 10.3390/bios15060355

**Published:** 2025-06-04

**Authors:** Minhyuk Lee, Minjae Lee, Sungjee Kim, Nokyoung Park

**Affiliations:** 1 Department of Chemistry, Pohang University of Science and Technology, Pohang 37673, Republic of Korea; lmh92@postech.ac.kr (M.L.); sungjee@postech.ac.kr (S.K.); 2 Chemistry and Nanoscience Major, College of Chemistry and Life Sciences, Myongji University, 116 Myongji-ro, Yongin-si 17058, Republic of Korea; lmj1594@mju.ac.kr

**Keywords:** stimuli responsiveness, pure DNA hydrogels, polymer hybrid DNA hydrogels, inorganic nanomaterial hybrid DNA hydrogels, bioanalysis, biomedicines

## Abstract

Hydrogels are three-dimensional network structures composed of hydrophilic polymers that can swell in water and are very similar to soft tissues such as connective tissue or the extracellular matrix. DNA hydrogels are particularly notable for biomedical applications due to their high biocompatibility, physiological stability, molecular recognition, biodegradability, easy functionalization, and low immunogenicity. Based on these advantages, stimuli-responsive DNA hydrogels that have the property of reversibly changing their structure in response to various microenvironments or molecules are attracting attention as smart nanomaterials that can be applied to biosensing and material transfer, such as in the case of cells and drugs. As DNA nanotechnology advances, DNA can be hybridized with a variety of nanomaterials, from inorganic nanomaterials such as gold nanoparticles (AuNPs) and quantum dots (QDs) to synthetic polymers such as polyacrylamide (PAAm) and poly(N-isopropylacrylamide) (pNIPAM). These hybrid structures exhibit various optical and chemical properties. This review discusses recent advances and remaining challenges in biomedical applications of stimuli-responsive smart DNA hydrogel-based systems. It also highlights various types of hybridized DNA hydrogel, explores various response mechanism strategies of stimuli-responsive DNA hydrogel, and provides insights and prospects for biomedical applications such as biosensing and drug delivery.

## 1. Introduction

Hydrogels are three-dimensional network structures composed of hydrophilic polymers that can swell in water and absorb more than 90% of their weight in moisture. Composed primarily of water, hydrogels possess a porous structure that facilitates the diffusion of molecules. In addition, their flexibility and structural similarity to soft tissues such as connective tissue or the extracellular matrix make them highly suitable for applications in tissue engineering and various biomedical fields [1,2]. The physical and chemical properties of hydrogels depend on the type and composition of the polymer used [3]. This property allows hydrogels to be designed and synthesized with tailored characteristics, such as in terms of network density, mechanical strength, and responsiveness to external stimuli [3]. In particular, responses to physical (such as temperature, electric or magnetic fields, light) and chemical (such as pH, ionic strength, and small or biological molecules) stimuli can be utilized in wound healing, drug delivery, biosensing, 3D printing, 3D cell culture, and bioactuators [4,5,6,7,8,9,10,11]. These stimuli-responsive hydrogels are in the spotlight as important functional materials in the fields of precision medicine, environmental solutions, soft robots, and tissue engineering as smart materials that respond to changes in the external environment and control their functions on their own, transcending the limitations of non-responsive structural materials [11,12]. DNA (deoxyribonucleic acid), a biopolymer, has received much attention as a material for composing stimuli-responsive hydrogels due to its various advantages, including specific molecular recognition, high biocompatibility, programmability, physiological stability, biodegradability, easy functionalization, and low immunogenicity [13,14].

DNA is a natural biopolymer consisting of four nucleotides: adenine (A), thymine (T), guanine (G), and cytosine (C), twisted into a double helix structure through Watson–Crick base-pairing interactions. The complementary base pairs between A and T (A–T) and G and C (G–C) play key roles in the structural stability and information storage function of DNA. DNA serves the role of storing and transmitting genetic information within living organisms, but with the development of DNA nanotechnology, it is attracting considerable attention as an ideal structural material for designing complex functional three-dimensional nanostructures [15]. DNA is chemically stable and can be easily synthesized to have the desired nucleotide sequence. These properties allow precise programming and realization of high-order nanostructures through self-assembly of DNA [15]. In addition, the specific molecular recognition ability of DNA for a wide range of targets from heavy metal ions to cells and reversible and dynamic structural transition ability make DNA an ideal candidate for stimuli-responsive hydrogels [16,17]. This structural transition capability of DNA hydrogels can be induced by changes in interactions between linker DNA strands forming the network, as well as by the folding or dissociation of specific DNA sequences in response to external stimuli. Such stimuli include changes in pH, temperature, target molecule concentration, or ion concentration, which can trigger reversible conformational changes or strand dissociation. Moreover, the porous structure of DNA hydrogels facilitates loading of various cargos such as therapeutics and signal generators, and their facile functionalization enables easy tailoring for specific applications. These properties allow DNA hydrogels to have particularly high applicability in biomedical fields, such as in drug delivery systems that enhance therapeutic efficacy and reduce side effects through controlled drug release and highly sensitive biosensors that enable early disease diagnosis by signal amplification [13,14,16,17]. However, DNA hydrogels made of pure DNA have limitations in that they have high preparation costs, low mechanical strength, and can be degraded by enzymes in organisms. One way to overcome these limitations is to prepare hydrogels hybridized with synthetic polymers such as polyacrylamide (PAAm) and poly(N-isopropylacrylamide) (pNIPAM) and inorganic nanomaterials such as gold nanoparticles (AuNPs) and quantum dots (QDs) by taking advantage of the fact that DNA can be easily conjugated with other functional materials [18,19,20,21].

**Figure 1:**
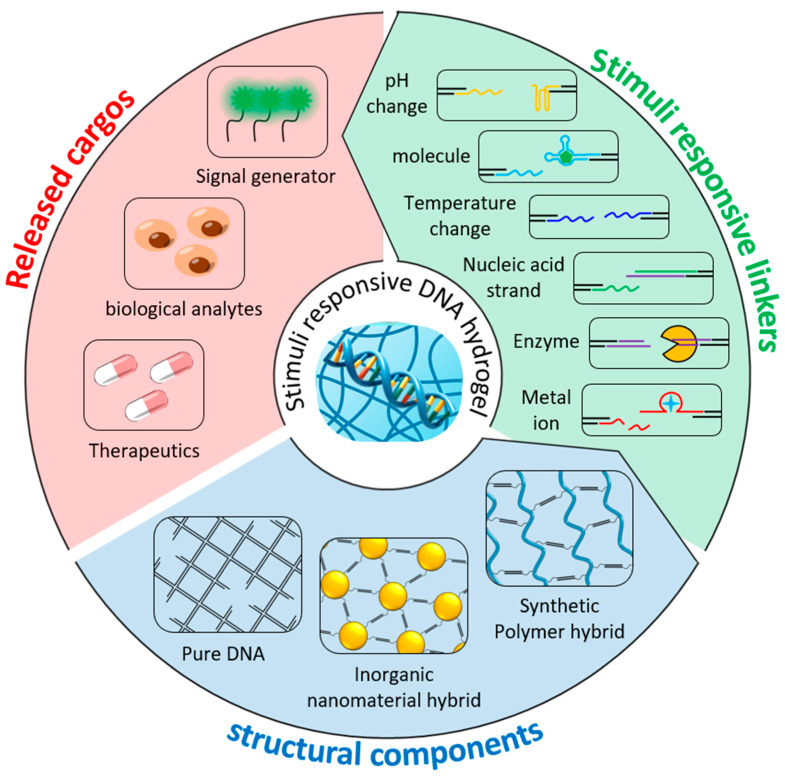
Overview of the composition of stimuli-responsive DNA hydrogels. Each component can be tailored for the intended purpose: pure DNA offers biocompatibility, while hybridization with inorganic nanomaterials or synthetic polymers provides additional stability and functionality. Stimuli-responsive linkers, selected based on target stimuli such as pH, temperature, or metal ions, induce structural changes that enable controlled release of therapeutics, analytes, or signal generators.

In this review, we comprehensively discuss recent research trends and remaining technological challenges for biomedical applications of pure DNA-based hydrogels and hybrid DNA hydrogels utilized as stimuli-responsive systems (Figure 1). In addition, we explore the working mechanisms and design strategies of DNA hydrogels in response to various chemical and physical stimuli, such as target recognition, structural transitions, and signal amplification, and present their potential and future prospects in biomedical applications, including biosensing and drug delivery systems. This review provides an overview of the current status of stimuli-responsive DNA hydrogel research and is expected to contribute to the advancement of the field by providing useful insights for establishing strategies for future technology development and clinical applications.

## 2. Pure DNA-Based Stimulation-Responsive DNA Hydrogel

Pure DNA hydrogel refers to a three-dimensional network structure composed solely of DNA without any hybridizing with other polymers or inorganic materials. Pure DNA hydrogels can be prepared by three main methods: hybridization between complementary sticky ends, enzyme-catalyzed ligation, and chain entanglement [22]. Enzymes called DNA ligase and DNA polymerase are involved in enzyme-catalyzed ligation and chain entanglement, respectively. In this section, we will introduce three main methods for preparing pure DNA hydration gels and discuss the design strategies and working mechanisms of stimuli-responsive DNA hydrogels prepared based on these methods.

### 2.1. Hybridization-Based DNA Hydrogel

**Figure 2:**
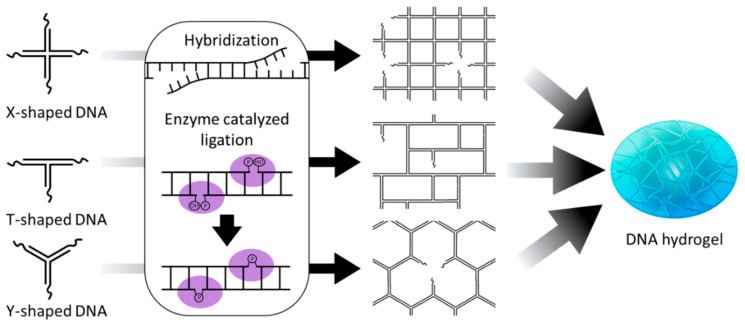
Schematic illustrations of pure DNA-based hydrogel formation through hybridization or enzyme-catalyzed ligation of branched DNA.

**Figure 3:**
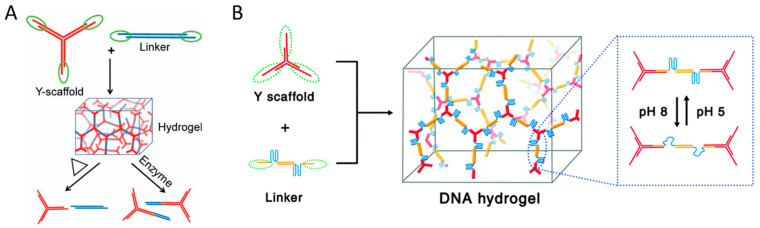
Hybridization-based stimuli responsive DNA hydrogel utilizing branched DNA. (**A**) Schematic image of a stimuli-responsive DNA hydrogel that exhibits structural transitions in response to enzyme and temperature changes (adapted from Ref. [23]). (**B**) Schematic of a stimuli-responsive DNA hydrogel that exhibits structural transitions in response to pH changes (adapted from Ref. [24]).

In DNA hydrogel preparation based on hybridization, a three-dimensional network is formed by hybridizing complementary sequences at their ends using branched DNA structures such as Y-shaped, X-shaped, and T-shaped DNA as building blocks (Figure 2) [23,24,25]. In this case, functionalized linker DNA with sequences complementary to branched DNA structures can be used together to give hydrogels a variety of functions [26]. For example, DNA molecules such as specific restriction sites, i-motifs, and aptamers, which respond selectively to external stimuli such as restriction enzymes, pH changes, and target molecules, can be employed as linkers to impart stimuli-responsive properties to DNA hydrogels. These hybridization-based cross-links are non-covalent interactions through hydrogen bonding of Watson–Crick base pairs, which enables dynamic and reversible structural transitions depending on salt concentration or temperature. This structural transition property, combined with stimuli-responsive DNA linkers, can be extended to precise and selective stimuli-responsive hydrogel systems. Xing et al. demonstrated a hydrogel system in which DNA strands containing recognition sites for the restriction enzymes EcoR I and BamH I were used as linkers, enabling effective sol–gel transitions through enzyme-catalyzed cleavage (Figure 3A) [23]. While this system induces highly specific sol–gel transitions because it is mediated by a specific enzyme-catalyzed reaction, the cleavage of DNA strands by the restriction enzymes results in irreversible gel dissolution. In contrast, Zhou et al. reported a DNA hydrogel capable of reversible structural transitions in response to pH changes using Y-shaped DNA in combination with linkers incorporating two i-motif structures (Figure 3B) [24]. Liu et al. further expanded this concept by constructing a DNA hydrogel using X-shaped DNA and linear DNA cross-linked via a single-stranded aptamer, which allowed for the specific capture and controlled release of thrombin [25]. These studies demonstrate that incorporating stimuli-responsive DNA motifs into linkers can be used to design DNA hydrogels whose sol–gel behavior can be programmed in response to various external stimuli.

Xing et al. also investigated the effect of sticky-end length on the sol–gel transition temperature and observed that increasing the number of hybridizing base pairs raised the transition temperature, suggesting that the sol–gel behavior can be tuned by adjusting the sticky-end length [23]. In this case, sensitivity to stimulation is determined by hybridization stability, which is controlled by the length of the complementary base sequence. Lai et al. optimized the two-step reaction system by tuning the hybridization length of the aptamer sequence [27]. In the first step, binding of adenosine induces the folding of the aptamer, resulting in the release of a complementary trigger DNA strand. In the second step, the released trigger DNA hybridizes with another aptamer sequence pre-bound to the target protein, leading to conformational change and subsequent release of the protein. In this study, a series of optimization experiments was performed on hybridization lengths ranging from 8 to 16 bp [27]. Based on the melting temperatures of the structures and efficiency of fluorescence recovery under adenosine exposure, 12–14 bp was identified as the optimal hybridization length. The melting temperature increased quickly with hybridization length up to 14 bp, after which the rate of increase slowed. However, beyond 14 bp, the duplex became overly stabilized, preventing adenosine-induced structural transitions. The trend of increasing stability of DNA duplexes with longer base pair lengths around 12 bp has also been observed in other studies [23,28,29]. There have also been reports demonstrating that sensitivity to a target can be tuned by adjusting the length of the strand hybridized to the aptamer or by modifying the aptamer sequence itself [30,31]. If the hybridized sequence is not sufficiently stable, nonspecific dissociation may trigger undesired structural transitions, and conversely, if it is too stable, strong complementary binding may prevent sufficient structural responses to the stimulus. Therefore, precise control of the length of the hybridization sequence is essential to design it to respond appropriately to specific stimuli.

**Figure 4:**
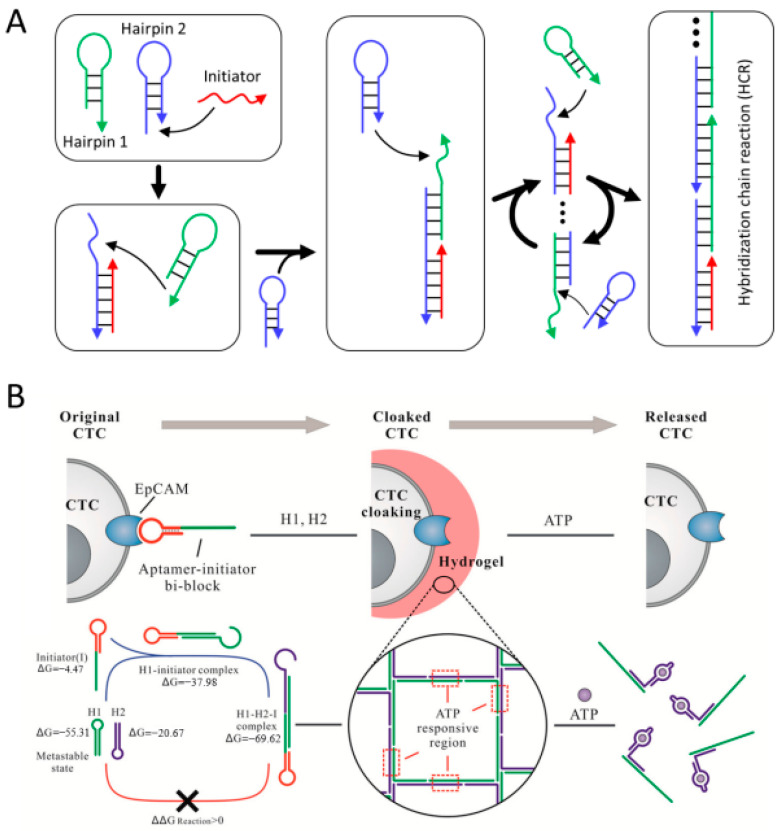
(**A**) Schematic image of the principle of HCR, a self-assembly reaction that proceeds sequentially by an initiator sequence. (**B**) Schematic image of the DNA hydrogel formation process for target cell captures via HCR induced by aptamer initiator binding to the cell surface (adapted from Ref. [35]).

The gelation process of hybridization-based DNA hydrogels does not require enzyme-catalyzed reactions, allowing them to be designed to undergo network formation and gelation from a dispersed solution state upon exposure to specific stimuli. In contrast to the aforementioned gel-to-sol transition, which undergoes reversible or irreversible gel-to-sol transition upon stimulation exposure, this approach involves DNA components initially present in a dispersed state that form a cross-linked gel network in response to specific stimuli [32,33,34,35]. The gel-to-sol system is advantageous for smart drug delivery and biosensor applications because it loads drugs and signal reporter into a 3D network structure and is rapidly dissolved and released by external stimuli, whereas sol-to-gel systems are advantageous for applications in on-demand assembly and target separation. This type of gel network formation mainly uses a hybridization chain reaction (HCR) mechanism in which two or more hairpins participate in the reaction (Figure 4A). HCR is a nonenzymatic self-amplifying DNA reaction induced by an initiator strand proposed by Pierce and Dirks [36]. The basic principle of HCR is that hairpins 1 and 2, which have complementary sequences, undergo a chain of strand displacement reactions by the initiator strand to form long linear or 3D network DNA structures.

Wang et al. reported a system to form macroscopic DNA hydrogels via HCR reaction induced by initiator strands [32]. Xu et al. extended this approach by designing a system that can detect target DNA without a separate label by generating a hydrogel only when target DNA is present [33]. Because this system can flexibly change the target DNA sequence, it has high potential for application in the diagnosis of various diseases by detecting circulating tumor DNA, viral DNA, or RNA. Dong et al. developed such a system by utilizing the target-triggered polymerization (TTP) mechanism of branched DNA [34]. They introduced target DNA as a linker to induce the self-assembly of two types of X-shaped DNA structures, enabling the discrimination of even single-base variations without the need for additional labeling. However, unlike HCR, this system does not allow a single trigger strand to induce repetitive self-assembly. Song et al. designed a capture–release system based on a stimuli-responsive DNA hydrogel formed by inducing HCR using aptamers that specifically recognize tumor cell surface proteins as initiators to isolate circulating tumor cells (CTCs) without damage (Figure 4B) [35]. The stimuli-responsive DNA hydrogel formed undergoes a gel-to-sol structural transition upon the addition of ATP, thereby enabling the effective release of the captured CTCs. This mechanism can be applied not only to DNA hydrogel formation but also to various signal amplification technologies.

### 2.2. Enzyme-Mediated DNA Hydrogel

In the case of DNA hydrogel preparation based on enzyme-catalyzed ligation, branched DNA building blocks first hybridize through complementary sticky arms to form a loose network, and then DNA ligase covalently links these prehybridized regions to produce a DNA hydrogel (Figure 2) [37]. While this approach offers improved mechanical strength and structural stability compared to hybridization-based systems, it requires a more complex preparation process. Moreover, it lacks the ability to undergo reversible structural transitions. Therefore, due to its limited stimuli responsiveness, this type of DNA hydrogel is often combined with other stimuli-responsive materials [38,39]. Therefore, it is mainly applied in fields such as tissue engineering, where structural support is required, and in sustained drug release systems [40,41,42,43]. However, rather than functioning through sol–gel transition, this system holds great potential as a biologically responsive platform triggered by biological stimuli [43,44]. Park et al. developed a DNA hydrogel-based cell free protein expression platform in which biological stimuli, such as enzymes and cell lysates, trigger the expression of mRNA and proteins [44]. This DNA hydrogel is synthesized through an enzyme-catalyzed ligation reaction involving XDNA and plasmid DNA containing genetic information. The system increases the local concentration of the plasmid and protects it from DNase degradation, resulting in significantly higher mRNA and protein expression levels compared to free plasmid systems. This platform technology was further developed into a nanoscale DNA hydrogel that can be delivered into cells, functioning as a nanoreactor for intracellular gene expression [45]. This nanoreactor was synthesized via enzyme-catalyzed ligation of XDNA, with one of its four arms having a blunt end and a plasmid DNA encoding shRNA designed to silence a mRNA of target gene. The resulting DNA hydrogel, loaded with T7 RNA polymerase, was delivered into cells, where it successfully expressed shRNA and suppressed the target gene.

**Figure 5:**
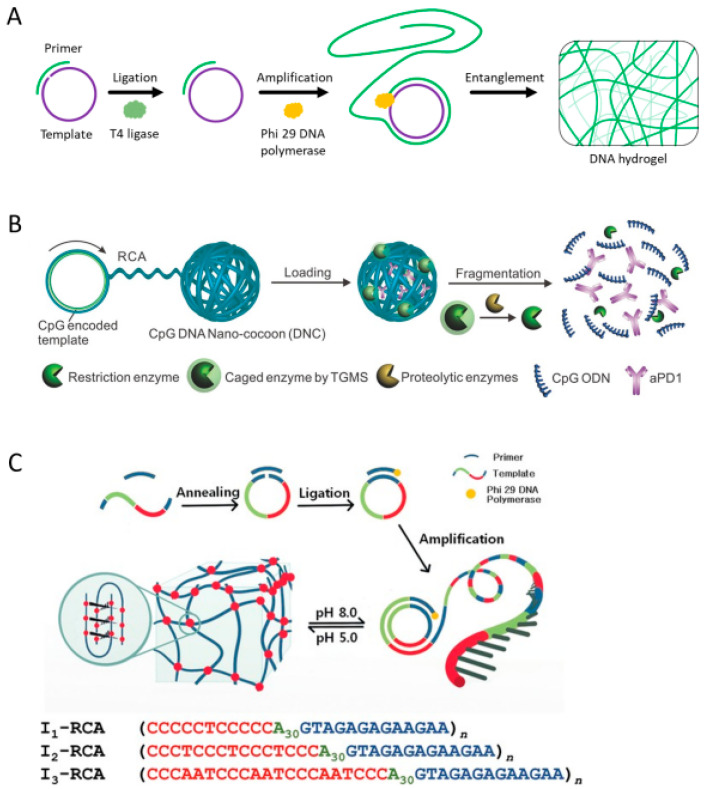
(**A**) Schematic image showing hydrogel formation by physical entanglement of long ssDNA produced by RCA. (**B**). Schematic image of RCA-based DNA hydrogel for co-delivery of CpG ODN and anti-PD-1 antibody for cancer immunotherapy (adapted from Ref. [48]). (**C**). Schematic image of pH-responsive DNA hydrogel formation through an RCA reaction using a template containing i-motif (adapted from Ref. [52]).

Another enzyme-mediated DNA hydrogel formation involves the physical entanglement and cross-linking of long single-stranded DNA (ssDNA) produced by rolling circle amplification (RCA) [46]. As mentioned above, the production of DNA materials, including pure DNA hydrogel, requires high costs. However, RCA provides a cost-effective, high-efficiency approach to synthesize large quantities of ssDNA through enzyme-catalyzed isothermal amplification [47]. The RCA reaction requires a circular DNA template, which is prepared by hybridizing a primer with a linear template DNA followed by an enzyme-catalyzed ligation reaction (Figure 5A). Phi 29 DNA polymerase then binds to the primer and continuously synthesizes a long ssDNA composed of tandem repeats complementary to the template. The generated long ssDNA forms a hydrogel network by self-hybridizing or physically entangling complementary sequences. RCA-based DNA hydrogels can be applied in various fields such as drug delivery, soft robotics, and biosensing [48,49,50]. By incorporating specific functional sequences, such as restriction enzyme cleavage sites, DNAzymes, i-motifs, or aptamers, into the template DNA, these functional domains are repeatedly incorporated into the DNA hydrogel generated via the RCA reaction [48,51,52,53]. Wang et al. reported a bioresponsive immunotherapy delivery system via RCA-based DNA hydrogel to suppress post-surgical tumor recurrence (Figure 5B) [48]. This DNA hydrogel is prepared by self-assembly of long ssDNA synthesized via RCA reaction from a template containing a CpG oligodeoxynucleotide (CpG ODN) sequence and a restriction enzyme cleavage site that induces an antitumor immune response. The DNA hydrogel was also loaded with a restriction enzyme that was activated in an inflammatory environment and an immune checkpoint inhibitor, anti-PD-1 antibody. Upon exposure to the post-surgical inflammatory environment, the activated restriction enzyme triggered dissolution of the hydrogel, resulting in the co-release of CpG ODN and anti-PD-1 antibody to promote a robust anti-cancer immune response. Xu et al. proposed a simple approach to prepare pH-responsive DNA hydrogels by incorporating i-motif sequences into the template (Figure 5C) [52]. They prepared DNA hydrogels with different mechanical strength and pH response properties by utilizing three types of i-motif (I1, I2, and I3) with different sequences. For example, compared to I1, which had limited pH reactivity, I3 showed faster and stronger pH reactivity, and I2 exhibited relatively excellent mechanical strength. As in the example above, a stimuli-responsive DNA hydrogel can be easily produced by including a specific sequence in the template. In addition to pure DNA hydrogels, functional DNA hydrogels can be prepared using functionalized primers or nucleotides during the RCA reaction or by introducing functional linkers after RCA to perform cross-linking [49,54,55,56].

**Figure 6:**
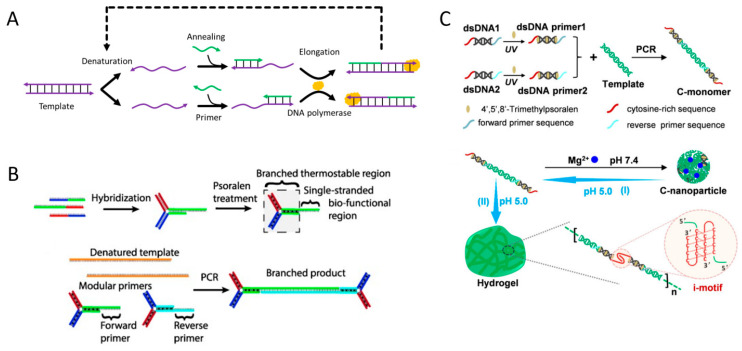
(**A**) Schematic image showing the three steps of PCR process. (**B**) Schematic image showing the process of forming thermostable branched PCR products for subsequent DNA hydrogel formation by PCR using YDNA containing primers (adapted from Ref. [58]). (**C**) Schematic image showing the formation of organelle-like hydrogels with self-assembling capability in acidic environments via PCR using primers containing i-motif sequences (adapted from Ref. [60]).

DNA polymerase-catalyzed polymerase chain reaction (PCR) reactions can also be used to prepare DNA hydrogels. PCR is a nucleic acid amplification technique invented by Mullis. It is a method to selectively copy and amplify a desired sequence by using a primer complementary to a specific part of template DNA [57]. The PCR process consists of three repeating steps: denaturation, annealing, and elongation (Figure 6A). In the denaturation step, heat is applied to separate the double-stranded template DNA. During annealing, primers bind to specific sequences on the template. Finally, in the extension step, DNA polymerase extends the primers by synthesizing complementary DNA strands along the template. DNA hydrogel formation involving a PCR reaction is a method that utilizes the DNA itself amplified during the PCR process as a component for structure formation [58,59,60,61]. Hartman et al. synthesized thermostable branched PCR products using primer-containing YDNA and used them to form DNA hydrogels (Figure 6B) [58]. Chen et al. reported a clathrate DNA hydrogel sensor system prepared by cross-linking tetrahedral DNA with PCR products, enabling the visual detection of *Salmonella* species [59]. This DNA hydrogel was synthesized using tetrahedral DNA with four arms as building blocks and PCR products, derived from the *Salmonella* genome, as linkers. AuNPs were loaded into the DNA hydrogel for visual detection. Guo et al. reported an i-motif DNA-based dynamic system in which PCR products containing C-rich sequences undergo structural transition into intracellular, organelle-like hydrogels intertwined as a network, mediated by the acidic environment of lysosomes (Figure 6C) [60]. They used dsDNA with C-rich sequences and primers that protruded from both ends to produce C-monomers with C-rich sequences at both ends via PCR. The C-monomers self-assemble into DNA nanoparticles (C-nanoparticle) through Mg^2+^ ion-mediated condensation, and these nanoparticles dissociate in the acidic environment of lysosomes, triggering the formation of organelle-like DNA hydrogels via i-motif structure formation. As demonstrated in the examples above, PCR can be effectively used to amplify DNA with specific sequences and structures, and the resulting PCR products can be utilized for the formation of DNA hydrogels. Moreover, PCR can also be directly used to form DNA networks. Finke et al. used PCR to form a DNA network composed of branched primers, and developed a functional DNA-based hydrogel system capable of cell-selective adhesion and release [61]. They used branched primers modified with peptides to selectively adhere to specific cells and formed a DNA hydrogel through PCR. The peptide-functionalized DNA hydrogel selectively adhered to target cells, and the attached cells were able to be released without damage following DNase treatment.

## 3. Hybrid DNA-Based Stimulation-Responsive DNA Hydrogel

Hybrid DNA hydrogels, formed by combining pure DNA materials with synthetic polymers or inorganic nanomaterials, represent an effective approach to enhance mechanical strength, in vivo stability, and cost-effectiveness. Moreover, incorporating materials with diverse properties can endow DNA hydrogels with additional responsiveness to various external stimuli [62,63]. In this section, we will introduce stimulus-responsive hybrid DNA hydrogels combined with synthetic polymers or inorganic nanoparticles and discusses their design strategies and working mechanisms.

### 3.1. Synthetic Polymer Hybrid DNA Hydrogel

Pure DNA hydrogels consist of DNA as both the backbone and the linker, whereas in synthetic polymer hybrid DNA hydrogels, the synthetic polymer serves as either the backbone or the linker. Synthetic polymer hydrogels have high structural stability, but limited reactivity and molecular recognition ability, whereas DNA hydrogels have specific molecular recognition ability and reversible and diverse stimuli responsiveness, but low structural stability [17]. To combine the advantages of both materials, high structural stability with precise and diverse stimuli responsiveness, synthetic polymers are generally used as the backbone, while DNA serves as the cross-linker. Synthetic polymer hybrid DNA hydrogels have been actively studied in various fields since the first PAAm hybrid DNA hydrogel was reported in 1990 [64]. PAAm, when hybridized with DNA, enhances structural stability, is cost-effective, exhibits low cytotoxicity, can be easily cross-linked with acrydite-modified DNA, and is readily processed into various forms such as films and microcapsules, making it one of the most extensively studied hybrid DNA hydrogel systems. Nagahara and Matsuda reported two PAAm hybrid DNA hydrogels by modifying DNA with acrydite. Acrydite is a functional group that can introduce an acrylate group to the 5′ end of DNA, thereby covalently anchoring DNA to a PAAm backbone. They proposed a method to fabricate PAAm-based hybrid DNA hydrogels in two ways: utilizing oligodeoxyadenylate (oligoA) as a cross-linking agent between PAAm hybrid oligodeoxythymidylate (oligoT-PAAm) or hybridizing oligoA–PAAm and oligoT–PAAm. This approach enabled reversible sol–gel transitions in PAAm. Lin et al. successfully demonstrated a sol–gel transition in PAAm-based hybrid DNA hydrogels by utilizing strand displacement reactions, taking advantage of the unique properties of DNA cross-linkers [65]. Synthetic polymer hybrid DNA hydrogels can further expand their application scope by imparting additional functions through synthetic polymers [66].

**Figure 7:**
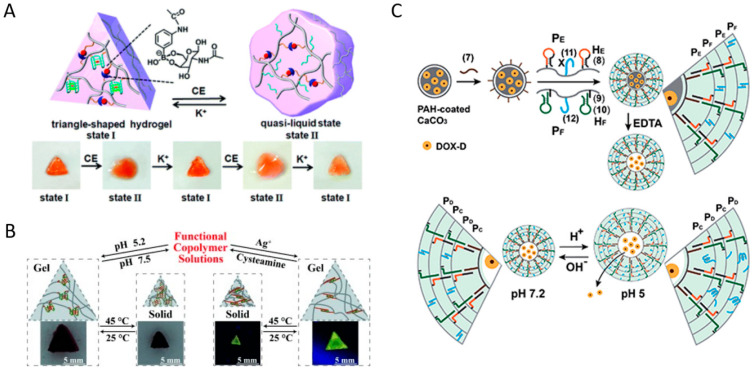
(**A**) Schematic image showing the working process of hybrid DNA hydrogels with reversible shape-memory structural transition properties by utilizing aminoglucose-boronate ester and K^+^-mediated G-quadruplex as synergistic cross-linkers (adapted from Ref. [68]). (**B**) Schematic image showing the working process of multi-stimulatory hybrid DNA hydrogels with reversible three-way conformational transition properties using thermoresponsive pNIPAM and pH-responsive i-motif as synergistic cross-linkers (adapted from Ref. [69]). Schematic image showing the working principle of pH-responsive microcapsules surface-coated with hybrid DNA hydrogel containing i-motifs via HCR. (**C**) (adapted from Ref. [70]).

Willner’s group went one step further and reported synthetic polymer-based hybrid hydrogels that exhibited dynamic structural transitions in response to various stimuli by incorporating stimuli-responsive DNA linkers into various polymer chains such as pNIPAM, poly(allylamine hydrochloride), and poly(acrylic acid), including PAAm [67,68,69,70]. They developed shape-memory and self-healing functional hybrid DNA hydrogels with variable mechanical stiffness by utilizing aminoglucose–boronate esters (type I) and stimuli-responsive nucleic acid units (type II) as synergistic cross-linkers (Figure 7A) [68]. In this system, since the type I linkage is non-responsive to stimuli, the hybrid DNA hydrogel does not fully dissolve. Instead, by incorporating DNA units that form G-quadruplexes in the presence of K^+^ ions, a reversible system was developed in which the hydrogel exhibited increased stiffness when K^+^ was present and reduced stiffness upon the removal of K^+^ by crown ether (CE). In addition, they utilized stimuli-responsive polymers and DNA units as synergistic cross-linkers to realize a synthetic polymer hybrid DNA hydrogel that reversibly responded to two stimuli (Figure 7B) [69]. pNIPAM is a thermoresponsive polymer that is hydrophilic below about 32 °C and becomes hydrophobic and shrinks above this temperature. Due to this property, it is known to exhibit a reversible sol–gel structural transition depending on temperature changes. They synthesized pNIPAM hybrid DNA hydrogels capable of structural transitions among solution–hydrogel–solid by introducing a pH-responsive i-motif or a DNA unit forming metal-assisted base pairing into temperature-responsive pNIPAM. For example, the system with i-motif introduced showed solid state at 45 °C, hydrogel state at 25 °C, and completely dissolved behavior above pH 7.5. Interestingly, while pNIPAM alone exhibits only a gel-to-solid transition, the incorporation of DNA enables more diverse structural transitions. Du et al. reported a pNIPAM hybrid DNA hydrogel system that improved the turnover frequency of catalytic reactions by utilizing the reversible shrinkage and swelling function of pNIPAM according to temperature change [62]. They observed that by utilizing the swelling–shrinkage process of the hydrogel to promote the uptake of external substances and the release of internal products, the catalytic efficiency of the catalytically active DNA unit was enhanced by up to 390%. The structural transition properties of these synthetic hybrid DNA hydrogels further expand their potential applications as drug delivery systems capable of controlled release. Willner’s group developed an anticancer therapeutic strategy using DNA–polyacrylamide-based hydrogel microcapsules that respond to multiple stimuli as a drug delivery system (Figure 7C) [70]. These microcapsules were fabricated by encapsulating anticancer drug-loaded CaCO_3_ microparticles with a multilayer polymer film composed of poly(allylamine hydrochloride) and poly(acrylic acid), followed by deposition of an acrylamide–DNA hydrogel film containing stimulus-responsive DNA linkers via HCR. These DNA linkers form i-motif structures under acidic conditions, leading to dissolution of the hydrogel film and subsequent release of the loaded anticancer drug. In addition, synthetic polymer hybrid DNA hydrogels can be formed through various types of interactions with synthetic polymers such as polyethylene glycol (PEG), polyethyleneimine (PEI), and poly(lactic-co-glycolic acid) (PLGA) [71,72,73].

### 3.2. Inorganic Nanomaterial Hybrid DNA Hydrogel

**Figure 8:**
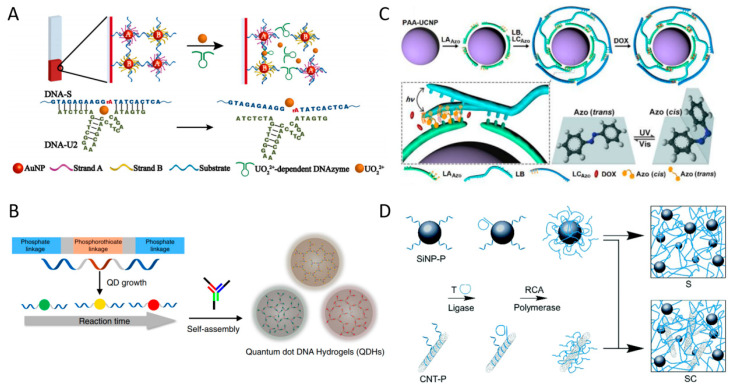
(**A**) Schematic image showing the AuNP-hybrid DNA hydrogel film sensor for detecting target metal ions (adapted from Ref. [75]). (**B**) Schematic image showing the formation of QD-hybrid DNA hydrogel through self-assembly of DNA-functionalized QDs and YDNA (adapted from Ref. [76]). (**C**) Schematic image showing UCNP-hybrid DNA nanopump that can effectively release loaded drugs in response to light (adapted from Ref. [78]). (**D**) Schematic image showing the production process of SiNP–CNT-hybrid DNA hydrogel via RCA reaction of SiNPs and CNTs functionalized with primers (adapted from Ref. [79]).

One of the major advantages of DNA is its ability to easily hybridize with a wide range of nanomaterials, which has led to the development of various hybrid DNA hydrogels in addition to polymers [74]. Functional inorganic nanoparticles such as magnetic nanoparticles (MNPs), AuNPs, QDs, and upconversion nanoparticles (UCNPs) are highly attractive candidates for constructing inorganic nanomaterial hybrid DNA hydrogels with diverse functionalities [49,63,75,76,77,78,79,80]. Tang et al. proposed a DNA hydrogel system hybridized with Fe_3_O_4_-based MNPs to fabricate a soft robot capable of navigating through narrow and complex pathways [49]. In general, DNA hydrogels possess low mechanical strength, making them suitable for soft robot fabrication. The ultrasoft magnetic DNA hydrogel reported in this study exhibited an elastic modulus of less than 1 Pa. MNPs typically have a diameter of less than 100 nm, are stable in aqueous solutions, and can be easily surface-functionalized with DNA or polymers, making them highly preferred nanomaterials for biomedical applications [81]. Here, the hybridized MNPs act as permanent cross-linking points within the DNA hydrogel, while the entanglement of RCA-amplified DNA chains serves as dynamic cross-linking points, enabling a magnetically guided soft robot system with a high shape-adaptive property. AuNPs are another nanomaterial that have been widely applied in a wide variety of fields, including biomedical fields, due to their excellent biocompatibility, unique optical properties, and easy surface modification properties [82]. Their most unique properties are their optical properties, which can be tuned from the visible to near-infrared range depending on their size and shape, and their photothermal effect, which allows them to absorb light and generate heat. Yata et al. functionalized the surface of gold nanoparticles with DNA and assembled them with hexapodna, a hexagonal DNA structure containing CpG motifs, via a self-assembly process to prepare a AuNP-hybrid DNA hydrogel capable of tumor photothermal immunotherapy [63]. This AuNP-hybrid DNA hydrogel releases hexapodna upon laser irradiation, while the photothermal effect of AuNPs induces heat shock proteins (HSPs) in cells through thermal stress. These hexapodna and HSPs stimulate immune cells, promoting the release of pro-inflammatory cytokines and the production of interferon γ. Through this series of immune responses, tumor growth was effectively suppressed. AuNPs can be used as effective probes for colorimetric analysis, not only for photothermal therapy but also due to their strong absorbance at specific wavelengths resulting from surface plasmon resonance. Liu et al. developed a colorimetric biosensing system based on an AuNP-hybrid DNA hydrogel for the selective detection of specific metal ions (Figure 8A) [75]. They fabricated a hydrogel film on a glass surface and implemented a colorimetric metal ion sensing system in which a linker containing a DNAzyme domain was activated in the presence of target metal ions such as Pb^2+^ or UO_2_^2+^, the hydrogel dissolved, and AuNPs were released to generate a colorimetric signal. The irreversible sol–gel structural transition by linker cleavage in this sensing system provided precise signal amplification output in the sensor, and the thin film structure enabled short detection times.

Quantum dots (QDs), widely used as fluorescence signaling generators in biosensing fields, are semiconductor nanoparticles on the nanometer scale that exhibit unique optical and electronic properties due to quantum confinement effects. Their emission wavelengths can be tuned by size, and they possess distinct advantages such as high resistance to photobleaching, excellent color purity, and high photoluminescence quantum yield (PLQY) [83]. As the application of quantum dots as biosensors has increased, various surface modification techniques have been developed to facilitate their use in biological systems [84]. Zhang et al. developed QD-hybrid DNA hydrogels (QDHs) that could be utilized for drug delivery and biosensing through self-assembly of DNA-functionalized QDs and YDNA (Figure 8B) [76]. They utilized DNA linkers containing phosphorothioate domains capable of binding to the QD surface to prepare DNA-functionalized QDs, and designed QD-hybrid DNA hydrogels (QDHs) to undergo enzymatic degradation within cells. Furthermore, by loading the QDHs with DOX, incorporating aptamers for specific cell targeting, and introducing siRNA for gene silencing, they implemented a multifunctional QD-hybrid DNA hydrogel system. In addition, optical sensor systems based on fluorescent nanomaterials such as QDs are primarily utilized in turn on/off detection systems, where fluorescence signals are regulated through energy transfer interactions with quenchers depending on the presence of target analytes [85]. This on/off approach offers the advantage of high sensitivity and accuracy due to its low background signal. Zhang et al. designed an aptamer-functionalized pure DNA hydrogel incorporating gold nanoparticles as quenchers and PEI-coated QDs as fluorophores to develop a biosensor platform targeting thrombin for protein detection [86]. AuNPs are well-known quenchers that non-radiatively accept energy from nearby fluorophores, effectively suppressing their fluorescence. In this system, they serve not only as quenchers to switch the fluorescence of QDs on and off but also as an indicating agent for colorimetric analysis. In contrast, Liu et al. developed a multiplexed sensing system capable of simultaneously detecting multiple target analytes by employing two types of QDs and AuNPs, both functionalized with ssDNA, rather than simply encapsulating them within a DNA hydrogel [87]. These ssDNAs were designed to specifically hybridize with DNA linkers containing aptamer domains specific for adenosine and cocaine. In this system, detection occurs as the aptamer binds to its target and undergoes conformational folding, leading to the disassembly of the nanostructure and an increase in QD fluorescence. Additionally, QDs possess narrow emission peaks, which offer advantages for multiplexed analysis. Hong et al. reported an optical sensor system utilizing Black Hole Quencher 2 (BHQ2) dye as a quencher, in which the fluorescence signal of QDs is turned off in the presence of the target and turned on in its absence [88].

Another type of fluorescence inorganic nanoparticles with unique optical properties, UCNPs exhibit great potential for biomedical applications due to their excitation by deep tissue-penetrating near-infrared (NIR) light, high photostability, and tunable emission wavelengths [89]. UCNPs exhibit upconversion, a phenomenon in which two low-energy photons are sequentially absorbed and then emitted as a single higher-energy photon. Tang et al. reported a UCNP-hybrid DNA hydrogel formed by cross-linking long ssDNA, synthesized via RCA, with positively charged UCNP through electrostatic interactions [77]. This hybrid DNA hydrogel, incorporating aptamer domains, can be utilized for the selective isolation and protection of target cells. Due to the unique properties of the UCNPs designed to absorb NIR light and emit visible light, it was effectively used to protect target cells from NIR-induced damage. Zhang et al. developed a very interesting photoresponsive DNA–azobenzene nanopump by combining UCNPs, which absorb NIR light and emit blue and ultraviolet (UV) light, with photoisomerizable azobenzene molecules (Figure 8C) [78]. Azobenzene exhibits reversible photoisomerization, transitioning from trans to cis under UV light and from cis to trans under blue light. This property of azobenzene can be utilized to fabricate photoreactive DNA linkers. While trans-azobenzene-modified DNA maintains a stable hybridized structure, the cis form destabilizes the duplex, leading to dehybridization. They implemented a nanopump system by assembling azobenzene-modified DNA onto UCNPs and loading it with DOX to promote drug release. The DOX intercalated within the DNA structure was effectively released through repeated cycles of DNA hybridization and dehybridization triggered by light stimulation. Silica nanoparticles (SiNPs) or carbon nanotubes (CNTs) can also be used to form hybrid DNA hydrogel [79,80]. SiNPs can be easily surface-modified with various materials including DNA, and are widely used for the delivery of drugs and fluorescent dyes due to their high biocompatibility and large loading capacity due to their porosity [90]. Additionally, CNTs can be simply combined with polymers or DNA through adsorption and are being studied in various fields, including biomedicine, due to their excellent mechanical and electrical properties and biocompatibility [91]. Hu et al. reported a programmable drug delivery system based on SiNP–CNT-hybrid DNA hydrogel for targeted therapy (Figure 8D) [79]. They prepared SiNP–CNT-hybrid DNA hydrogels by utilizing RCA of primer-functionalized SiNPs and CNTs mixed with template DNA. In this system, SiNPs were loaded with fluorescent dyes for disease diagnosis, and the DNA hydrogel structure was used as a drug delivery carrier for loading DOX and incorporating specific molecular recognition domains for targeted therapy. Schipperges et al. also utilized RCA-based SiNP–CNT-hybrid DNA hydrogels [80]. They incorporated plasmid DNA into a bulk-sized hybrid DNA hydrogel and applied it to cell-free protein synthesis for the expression of mRNA and proteins in solution.

## 4. Stimuli-Responsive DNA Linker

**Figure 9:**
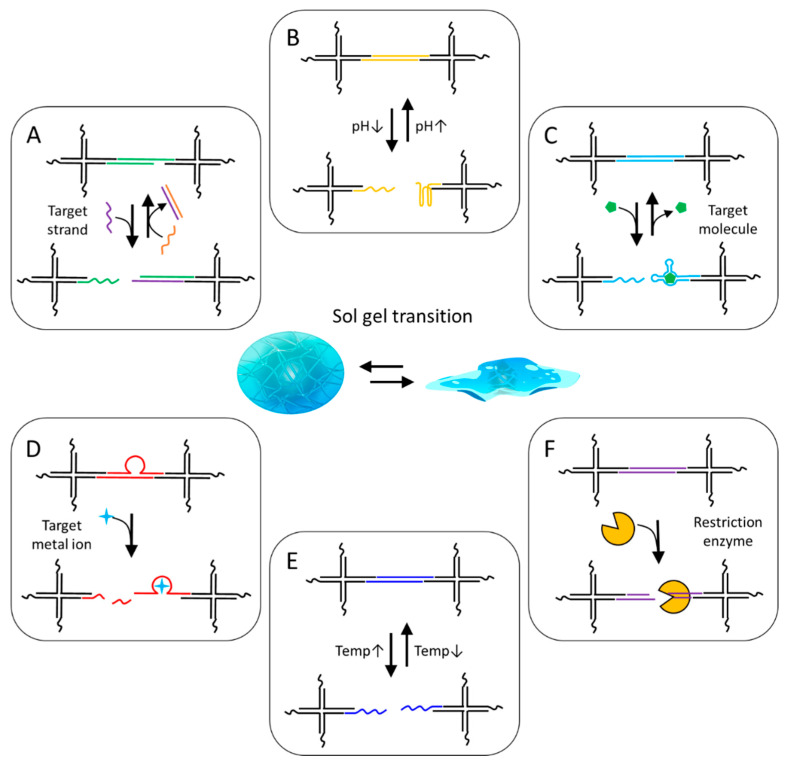
Schematic illustrations of stimuli-responsive DNA linkers. (**A**) Toehold-mediated DNA strand displacement induced by complementary nucleic acid strands. (**B**) pH-responsive i-motif C-quadruplex structure formation. (**C**) Target molecule-induced conformational change of aptamer. (**D**) Metal ion-responsive DNAzyme activation. (**E**) Temperature-responsive conformational change of DNA strands. (**F**) Sequence-specific cleavage by restriction enzymes.

As mentioned above, stimuli-responsive DNA linkers can be utilized to impart stimuli responsiveness to hybridization-based DNA hydrogels (Figure 9). These DNA linkers are generally short DNA molecules consisting of specific nucleotide sequences, which can induce reversible structural transitions of DNA hydrogels by folding and opening under specific conditions. DNA functional nanomaterials, such as aptamers, i-motifs, and DNAzymes, are chemically stable and can serve as effective linkers, with the added advantage of being programmable to selectively respond to diverse physical and biological stimuli through screening of random-sequence DNA libraries [92,93,94]. This section mainly introduces responsive DNA linkers and discusses their working mechanisms and applications.

### 4.1. Nucleic Acid Strand Responsive Linker

**Figure 10:**
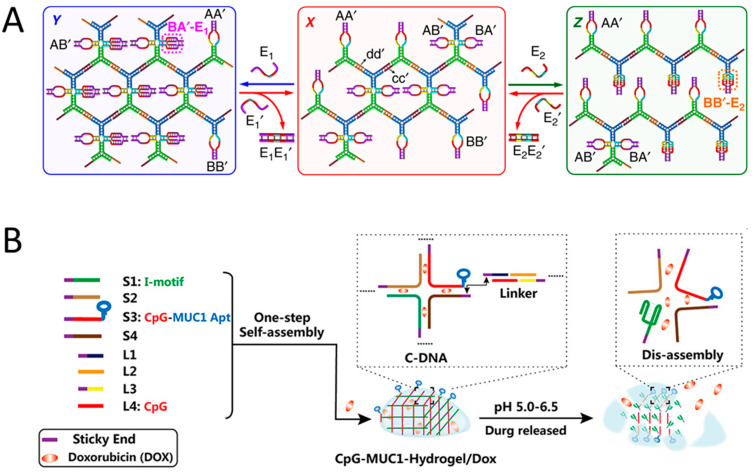
(**A**) Schematic image showing a DNA hydrogel capable of reversible transitions between three distinct structural stiffness states via two strand displacement reactions (adapted from Ref. [98]). (**B**) Schematic image showing a DNA hydrogel that releases drugs through pH-responsive structural transitions for cancer cell targeting (adapted from Ref. [103]).

DNA hydrogels that recognize and respond to specific nucleic acid targets can be prepared by incorporating complementary sequences as linkers (Figure 9A). The ability to recognize specific nucleic acid molecules and undergo structural transitions is based on the intrinsic property of sequence specific hybridization with complementary strands, enabling target specific strand displacement through toehold-mediated mechanisms. The structural transition can be reversibly regulated by adding a counter-target strand with a complementary sequence to the target strand. The rate of this reaction is correlated with the length of the toehold, up to six bases, suggesting that the complementary hybridization step of the toehold region determines the overall reaction rate [95]. Toehold-mediated strand displacement reactions can be applied to various systems, including dynamic molecular assembly and signal amplification mechanisms such as HCR, catalytic hairpin assembly (CHA) reaction, and DNA walker [32,33,35,96,97]. Yue et al. developed DNA hydrogels capable of reversible transitions between three different structural stiffness states using toehold-based strand displacement reactions (Figure 10A) [98]. A distinctive feature of this system is that it utilizes two trigger strands (E1 and E2) and two counter-trigger strands (E1’ and E2’) with complementary sequences to achieve a reversible three-state structural transition. E1 or E2 induces a structural transition in the intermediate stiffness state of DNA hydrogel via strand displacement, leading to increased or decreased stiffness, respectively. E1’ or E2’, bearing a complementary sequence, removes the E1 or E2 from the DNA hydrogel, thereby restoring it to its original stiffness state. Buchberger et al. reported a system to reversibly control the stiffness of gelatin hybrid DNA hydrogels by adding trigger strands [99]. Wang et al. developed a DNA hydrogel sensor for diagnosis of cancer by inducing sol–gel structural transition of DNA hydrogel through miRNA-induced toehold-based strand displacement reaction [100]. Hong et al. proposed an ultrasensitive detection system for target DNA using changes in fluorescence intensity based on the mechanism in which quencher YDNA, amplified through a cyclic reaction induced by target DNA, quenches the fluorescence of QDs [88]. In this system, quencher YDNA is amplified through a cyclic reaction initiated by a toehold-mediated strand displacement reaction with target DNA and self-assembles with ssDNA-QDs to form a QD–DNA hydrogel. The hydrogel formation quenches the fluorescence signal of QDs, thereby enabling the detection of the presence of target DNA.

### 4.2. pH Change-Responsive Linkers

DNA hydrogels that respond to pH changes can be prepared by introducing i-motif sequences as linkers (Figure 9B). An i-motif is an ssDNA structure composed of approximately 12 to 20 nucleotides that forms a C-quadruplex under acidic pH conditions [101]. This C-quadruplex is stabilized by interactions between protonated cytosine and deprotonated cytosine (C^+^-C). Because of these pH-responsive properties, i-motifs are mainly utilized in DNA nanomotors, intracellular pH sensors, and drug delivery systems targeting acidic tumor microenvironments [24,52,60,70,102,103]. Guo et al. reported a reversible system that enables transitions between quasi-liquid and hydrogel states using two types of acrydite-modified DNA strands [102]. The distinctive feature of this system lies in the integration of Watson–Crick base-pairing DNA, which maintains a loose structure regardless of pH, with C^+^–C base-pairing DNA that responds to low pH, enabling the construction of a pH-responsive shape-memory hydrogel. The i-motif that forms C-quadruplexes in acidic environments has great potential for implementing drug delivery systems that can be released in a controlled manner in low-pH disease environments. Wei et al. reported a DNA hydrogel that targets cancer cells using the MUC1 aptamer and releases drugs by undergoing structural transition through i-motif formation in lysosomes, which have an acidic environment after entering the cell (Figure 10B) [103]. They were designed to introduce an i-motif sequence to one of the four strands that make up XDNA to form a C-quadruplex in an acidic environment and thereby induce sol–gel structural transition. They also loaded doxorubicin, a representative anticancer drug that intercalates into DNA, into DNA hydrogel, and it was released into cells, as the DNA hydrogel dissolves in an acidic environment. DNA hydrogels have great potential as carriers of nucleic acid therapeutics (NATs) because they can be loaded with NATs such as antisense oligonucleotide (ASO), siRNA, plasmid DNA, and mRNA without additional conjugation steps and exhibit responsiveness to physiological or biological stimuli for controlled release [40,43,44,104,105]. Wu et al. reported a pH-responsive DNA hydrogel-based delivery system for ASO and DOX, utilizing dendritic DNA with three C-rich cross-linking branches and one loading branch [40]. This DNA hydrogel dissolves in the acidic tumor microenvironment, releasing ASO and DOX to exhibit anticancer effects. Similarly, Fu et al. reported a DNA hydrogel-based mRNA delivery system composed of XDNA and i-motif linkers, capable of loading enhanced green fluorescent protein (EGFP) mRNA and effectively releasing it within cells in response to the acidic pH of lysosomes [105]. They confirmed that the GFP mRNA, delivered via the DNA hydrogel and released inside the cells, was successfully translated into EGFP.

The i-motif-based reaction system can be expanded in function by combining it with other functional systems so that the reaction target responds to various stimuli, not just pH changes. Qin et al. achieved light-induced structural transitions of a DNA hydrogel by utilizing an i-motif in combination with a photoisomerizable merocyanine photoacid [106]. In this system, merocyanine is photoisomerized to spiropyran by light, releasing a proton, and when the light is blocked, it is converted back to merocyanine, changing the pH. Based on this mechanism, they constructed a photoresponsive structural transition system that utilizes light-controlled pH changes. Jeong et al. developed a DNA hydrogel-based capillary analysis platform that can detect short nucleic acid strands with high sensitivity by utilizing i-motifs [107]. The target nucleic acid strands amplify YDNA structures containing i-motif-forming sticky ends through CHA reaction, and self-assemble in an acidic environment to form DNA hydrogels. When the DNA hydrogel is formed, the capillary action of the solution is suppressed, so extremely small amounts of target nucleic acid strands can be sensitively detected. In this way, the i-motif can be applied not only to pH changes but also to a variety of stimuli.

### 4.3. Target Molecule-Responsive Linkers

**Figure 11:**
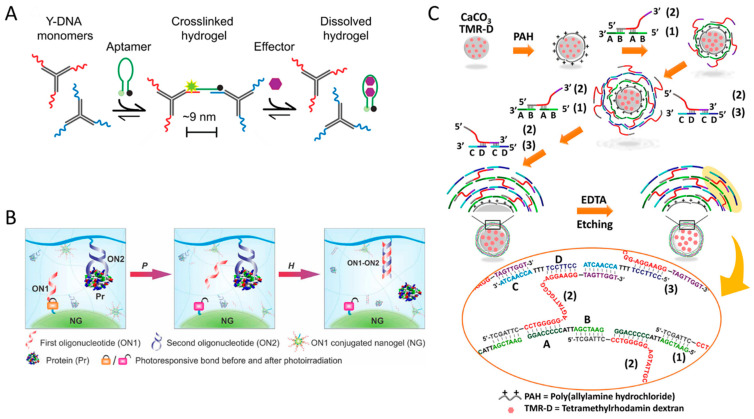
(**A**) Schematic image showing the sensing mechanism of a DNA hydrogel incorporating an adenine aptamer (adapted from Ref. [111]). (**B**) Schematic image showing the mechanism of protein capture by aptamers incorporated in DNA hydrogel and release by light irradiation (adapted from Ref. [113]). (**C**) Schematic image showing ATP-responsive drug release mechanism of DNA-based microcapsules (adapted from Ref. [115]).

DNA hydrogels that respond to a variety of target molecules, from small molecules to cell surface proteins, can be prepared by introducing sequences containing aptamers as linkers (Figure 9C). Aptamers are short nucleic acid strands generally consisting of 20 to 100 nucleotides that have a unique three-dimensional folded structure that exhibits high affinity for specific target molecules. Aptamers specifically interact with their target molecules through noncovalent interactions such as hydrogen bonding, electrostatic interactions, and shape complementarity [108]. Therefore, the aptamer contained in the linker constituting the DNA hydrogel can be used to bind to specific molecules and induce structural transition of the DNA hydrogel. Aptamers are molecular recognition elements that are functionalized into DNA hydrogels to target specific cells and can be used as drug delivery vehicles to enable effective targeted therapy [70,98,109,110]. Furthermore, aptamers incorporated into the linker constituting the hydrogel can also be used to selectively bind to specific molecules to induce structural transitions [35,111,112,113,114]. Simon et al. prepared an adenine-responsive DNA hydrogel by self-assembling an adenine aptamer, a linker, and Y DNA, and used it to analyze the responsiveness and reaction kinetics of adenine binding (Figure 11A) [111]. They functionalized quenchers and fluorophores at both ends of the aptamer and utilized them as linkers, and the fluorescent signal was emitted in the open structure formed by the DNA hydrogel. The fluorescent signal was utilized as an indicator based on the mechanism by which the DNA hydrogel dissolves and the fluorescence is quenched when the aptamer folds in the presence of adenine. Li et al. reported a DNA hydrogel-based sensor that can quantitatively analyze the concentration of a target substance by utilizing the principle that the target substance binds to an aptamer inside the DNA hydrogel, dissolving the hydrogel and thereby changing the flow velocity within the capillary [112].

As mentioned earlier, aptamer-based stimulus-responsive DNA hydrogels can be used to capture and release analytes such as proteins and cells, serving as a valuable platform for subsequent detailed analysis [25,35]. The release step involves introducing a nucleic acid strand with a higher affinity for the aptamer than the target molecule or using a process that dissolves the DNA hydrogel structure. Lai et al. developed a system that can precisely control this emission process by light irradiation (Figure 11B) [113]. This system utilizes two DNA oligonucleotides, the first oligonucleotide (ON1), which is conjugated with nanogels (NGs) via a photosensitive linker, and the second oligonucleotide (ON2), an aptamer covalently attached to the bulk hydrogel. In the capture phase, ON2 binds to the target protein, forming a protein–DNA complex. Upon light irradiation in the release phase, ON1 is released from the NGs and hybridizes with ON2, leading to the dissociation of the protein–DNA complex and the subsequent release of the protein. Aptamer-based responsive DNA hydrogels can also be utilized as logic gates or controlled-release drug delivery systems by designing them to release cargos in response to specific molecular targets [113,114]. Yin et al. implemented AND/OR logic gate functionality via a responsive DNA hydrogel system containing two aptamers targeting ATP and cocaine [114]. They prepared DNA hydrogels by copolymerizing two types of single-stranded DNA strands into polyacrylamide polymers and then hybridizing them with cross-linker DNAs. The hydrogels are loaded with gold nanoparticles (AuNPs), which serve as indicator reagents for visual signal detection when the hydrogels are dissolved by target molecules. For the AND logic gate, three DNA strands containing two aptamers are hybridized in a YDNA configuration, the hydrogel structure is maintained even if one of the two aptamers is folded, and it is designed to dissolve when both target molecules are present. For the OR logic gate, the cross-linker DNA is designed to contain two aptamer domains, and the hydrogel structure is designed to dissolve when one of the two aptamers is folded. Liao et al. reported DNA-based responsive microcapsules that release drugs in response to ATP (Figure 11C) [115]. These DNA microcapsules are formed by depositing multilayers of DNA containing ATP aptamers on CaCO_3_ microparticles preloaded with drugs, after which the CaCO_3_ cores are etched with EDTA. The aptamer contained in this DNA layer is incorporated into cancer cells overexpressing ATP, and when bound to ATP, it dissociates and releases the loaded drug. In a similar vein, Mo et al. developed an effective and target-specific drug delivery platform by loading doxorubicin, which intercalates into double-stranded DNA (dsDNA), into ATP-responsive DNA-based nanogels [116]. In this system, the drug release mechanism is the aptamer structurally folding in the presence of ATP, dissociating the DNA double helix and releasing the inserted Dox.

### 4.4. Metal Ion-Responsive Linkers

**Figure 12:**
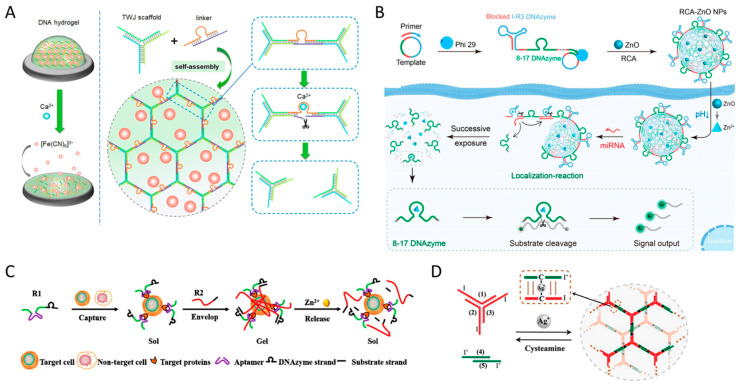
(**A**) Schematic image showing the Ca^2+^ ion detection mechanism using DNA hydrogel incorporating Ca^2+^-activated DNAzyme (adapted from Ref. [120]). (**B**) Schematic image showing a DNA hydrogel that combines two types of DNAzyme for miRNA detection and signal amplification (adapted from Ref. [123]). (**C**) Schematic image showing a DNA hydrogel incorporating aptamers and DNAzymes for selective capture and release of target cells (adapted from Ref. [125]). (**D**) Schematic image showing the formation of DNA hydrogel in the presence of Ag^+^ via metallo-base pairs (adapted from Ref. [128]).

DNA hydrogels that respond to metal ions can be prepared by introducing a sequence containing a DNAzyme as a linker (Figure 9D). DNAzyme is an ssDNA molecule that has a biological catalytic function, such as nucleic acid cleavage, using a metal ion as a cofactor [117]. DNAzymes, which catalyze substrate strand cleavage reactions, structurally have two arms at either end of a circular catalytic core that can bind to target nucleic acids. Since the sequence control of these binding arms has minimal impact on catalytic activity, it can be programmed and designed relatively freely depending on the sequence of the substrate strand [118]. In contrast, the catalytic core sequence directly affects the catalytic activity [119]. With the assistance of divalent metal ion cofactors bound to this region, a transesterification reaction is catalyzed, resulting in the cleavage of the substrate strand bound to the binding arms. Therefore, DNAzymes contained in the linker that constitutes the DNA hydrogel can be used to induce the structural transition of the DNA hydrogel by binding to a specific metal ion cofactor [75,120,121]. Chai et al. prepared a DNA hydrogel using a linker containing a DNAzyme domain that binds calcium (Ca^2+^) ions as a cofactor, and utilized it as an electrochemical biosensor that can sensitively and selectively detect Ca^2+^ ions. (Figure 12A) [120]. In this system, the presence of Ca^2+^ ion activates the DNAzyme, which cleaves the linker within the hydrogel, leading to the dissolution of the DNA hydrogel. The hydrogel encapsulates the electrochemical signal molecule [Fe(CN)_6_]^3−^, which is released upon hydrogel dissolution, resulting in an increase in the electrochemical signal. Huang et al. developed a lead (Pb^2+^) ion-responsive DNA hydrogel that can detect Pb^2+^ ions by utilizing a DNAzyme activated by Pb^2+^ ions and its substrate strand as a cross-linker [121]. In this system, AuNPs are loaded into the DNA hydrogel, and upon activation of the DNAzyme, the hydrogel dissolves, releasing the AuNPs for visual detection of lead ions. Additionally, by utilizing DNAzymes that are activated by Cu^2+^, Mg^2+^, and Zn^2+^ ions, a DNA hydrogel-based sensor that can specifically detect each metal ion can be prepared [122].

The DNAzyme system can be utilized not only for metal ion detection but also for detection of various target molecules and signal generation mechanisms [48,62,123,124,125]. Du et al. utilized the reversible shrinkage and swelling properties of pNIPAM hybrid DNA hydrogel to enhance the cleavage rate of DNAzymes, which was confirmed by the amplified fluorescence signal of reporter RNA with fluorophores and quenchers modified at both ends [62]. Shang et al. prepared DNA nanogels capable of detecting intracellular miRNAs via RCA from templates containing two DNAzyme domains (Figure 12B) [123]. The ZnO nanoparticles co-loaded within the DNA hydrogel release Zn^2+^ ions under acidic conditions, thereby activating the DNAzyme. The I-R3 DNAzyme hybridizes with the target miRNA and undergoes self-cleavage, leading to the dissolution of the DNA hydrogel. Another DNAzyme cleaves a reporter strand labeled with a fluorophore and quencher at both ends, generating and amplifying a fluorescence signal. Wang et al. reported a DNA hydrogel that integrates both aptamers and DNAzymes for sensitive detection of the marine biotoxin okadaic acid using Raman spectroscopy [124]. When the aptamer recognizes okadaic acid, the DNAzyme is released, causing the DNA hydrogel to dissolve, thereby releasing the SERS Raman signal tag and amplifying the detection signal. The structural transition of DNA hydrogels by these metal ion-dependent DNAzymes can be utilized for selective release of captured molecules or controlled release of drugs, as with other functional DNA linkers. Hou et al. reported a system for selective capture and release of target cancer cells using DNA hydrogels utilizing aptamers and DNAzymes synthesized by RCA (Figure 12C) [125]. This system consists of an R1 strand containing an aptamer and DNAzyme domains and an R2 strand containing the substrate domain. Initially, the R1 strand binds to the target cells in solution and the R2 strand hybridizes to the binding arms of the DNAzyme on R1, enabling three-dimensional capture of the target cells. Subsequently, the addition of Zn^2+^ ions activate the DNAzyme, inducing a sol–gel transition and releasing the captured cells. Wang et al. developed a DNA hydrogel-based anti-cancer therapy strategy in which a self-degradable DNAzyme activated inside cells triggers the release of a therapeutic DNAzyme that effectively silences tumor-associated genes [126].

In addition to using DNAzymes, metal-mediated base pairs (metallo-base pairs) can also be used to develop DNA hydrogel-based metal ion sensors. Metallo-base pairs are non-canonical nucleic acid base pairs, unlike Watson–Crick base pairs, that are formed by the coordination of a metal ion between two nucleotides. Hg^2+^-mediated thymine–thymine base pairs (T–Hg^2+^–T) and Ag^+^-mediated cytosine–cytosine base pairs (C–Ag^+^–C) are well known and have been widely applied in metal ion sensors and heavy metal trapping systems [69,127]. Guo et al. proposed a Ag^+^ ion detection sensor that forms C–Ag^+^–C base pairs and forms a DNA hydrogel in the presence of Ag^+^ using YDNA with a toehold sequence including a C–C mismatch (Figure 12D) [128]. After treatment with cysteamine, the Ag^+^ ions were removed, leading to the dissolution of the hydrogel, and the reversible sol–gel transition was successfully demonstrated. They also extended this system to a PAAm-hybrid DNA hydrogel and observed structural transitions of the hydrogel at lower concentrations of Ag^+^ ions. This is because fewer DNA bases are required to form the same volume of hydrogel in the hybrid DNA hydrogel system, suggesting that hybrid DNA hydrogels are more cost-effective than pure DNA hydrogels. Lastly, they expanded the system to a pNIPAM-hybrid DNA hydrogel and reported a triple-phase structural transition—solid, hydrogel, and solution—in response to dual stimuli of pH and Ag^+^ ions [69]. Similar to these C–Ag^+^–C based structural transfer systems, Gao et al. proposed a microfluidic chip sensor for detecting Hg^2+^ ions using a stimulus-responsive DNA hydrogel containing T-rich sequences that shrink in the presence of Hg^2+^ ions [129].

### 4.5. Temperature-Responsive Linkers

Heat-responsive DNA hydrogels can be prepared by introducing dsDNA hybridized as a linker without any additional functional domains (Figure 9E). The double-stranded structure of DNA is stabilized by hydrogen bonds between complementary base pairs, and these non-covalent bonds are sensitive to heat. As the temperature increases, the hydrogen bonds gradually weaken, eventually leading to strand separation. In fact, several studies have reported cases in which DNA hydrogels using hybridized DNA as a cross-linking agent changed their structural properties in response to heat [23,130]. As mentioned above, the melting temperature of DNA is determined by the number of base pairs [23]. According to this study, increasing the base pair length of the linker from 8 bp to 12 bp raised its melting temperature from 39.6 °C to 53.5 °C. Additionally, the study observed that when one mismatch is included in the 8 bp base pair length, the melting temperature significantly decreases to 20.8 °C. These results suggest that the hybridization stability of the DNA linker is a key factor in determining the melting temperature of the DNA hydrogel. The hybridization stability is also affected by GC content as well as base pair length [131]. This is because G-C pairs form three hydrogen bonds compared to two hydrogen bonds for A-T pairs, making GC-rich sequences more thermally stable interactions. Zhou et al. measured the melting temperature of a pH-responsive hydrogel containing an i-motif structure and observed values of 44 °C and 42 °C at pH 5.0 and 8.0, respectively [24]. Interestingly, the linker itself exhibited a melting temperature of 49 °C, suggesting that the C-quadruplex formation of the i-motif affects the hybridization stability of the linker. In summary, the temperature responsiveness of DNA hydrogels can be modulated through various interactions, including salt concentration and the hybridization stability of the linker strands.

**Figure 13:**
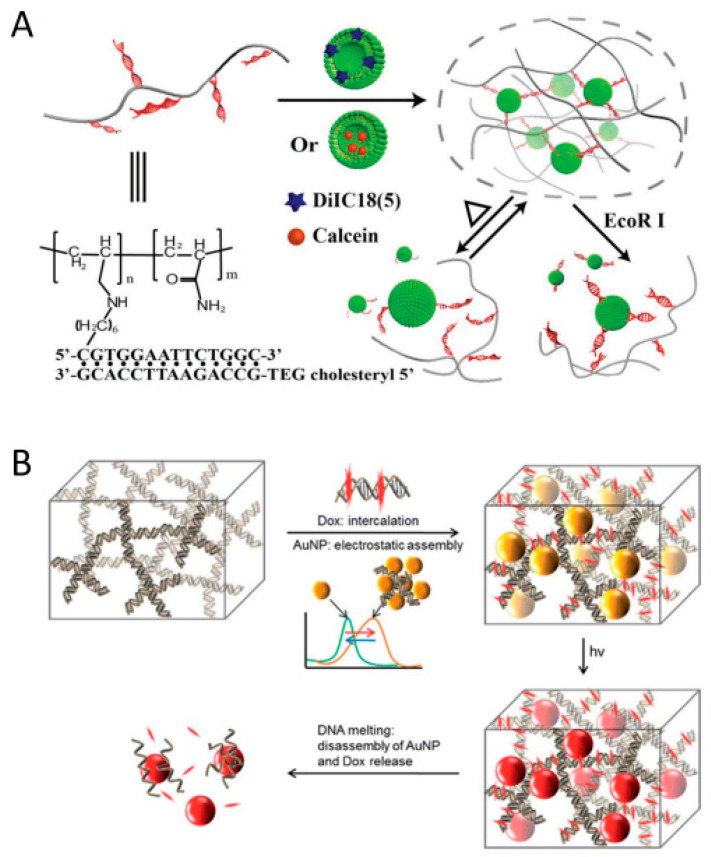
(**A**) Schematic image showing a DNA hydrogel-based drug delivery system in which drug release is triggered by temperature changes or restriction enzymes (adapted from Ref. [130]). (**B**) Schematic image showing the mechanism of drug release by light irradiation from DNA hydrogel loaded with DOX and AuNPs (adapted from Ref. [132]).

Since temperature-responsive DNA hydrogels dissolve upon increasing temperature, they can be effectively utilized in drug delivery systems. Lyu et al. reported a DNA hydrogel-based drug delivery system in which sol–gel transitions are induced by temperature changes or restriction enzymes to release the drug (Figure 13A) [130]. This temperature-responsive DNA hydrogel is prepared by hybridizing cholesterol-modified ssDNA with acrydite-modified ssDNA, forming cross-links with drug-loaded liposomes through hydrophobic interactions. Subsequent temperature changes or enzymatic treatment induces the dissociation of DNA linkers, leading to the release of the liposomes. AuNPs, a representative type of plasmonic metal nanoparticle, are known to convert absorbed light into heat energy through a thermal relaxation process and then emit it to the outside [82]. Therefore, incorporating AuNPs into a DNA hydrogel enables the system to respond to light, as the heat generated upon light irradiation induces the dissolution of the hydrogel. Song et al. reported a photo-responsive anticancer drug delivery system by loading AuNPs into Dox-intercalated DNA hydrogels (Figure 13B) [132]. Positively charged AuNPs are loaded through electrostatic interactions with negatively charged DNA hydrogels. This system provides the additional advantages of shifting the absorption region to the near infrared with higher tissue penetration depth, and the DNA hydrogel dissolves upon light irradiation, allowing the dispersed AuNPs to be rapidly removed from the body. Conversely, a system has also been proposed to form DNA hydrogels by generating local heat by irradiating them with light. Shimomura et al. reported a method to prepare microscale DNA hydrogels with flexible and tunable shape control using patterned light irradiation [133]. They used short ssDNA with both ends functionalized with Black Hole Quencher 1 (BHQ1) as a blocking strand to prevent the sticky ends of YDNA from cross-linking. Upon light irradiation, localized heat is generated at BHQ1, which induces the dissociation of the blocking strand. Subsequently, the activated sticky ends of YDNA hybridize with linker DNA, leading to the formation of a DNA hydrogel.

### 4.6. Restriction Enzyme-Responsive Linkers

**Figure 14:**
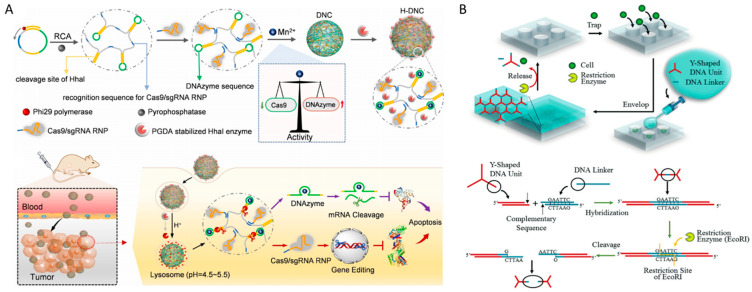
(**A**) Schematic image showing the anti-cancer therapeutic mechanism of DNA hydrogel that simultaneously releases Cas9/sgRNA RNP and DNAzyme by a restriction enzyme activated at acidic pH (adapted from Ref. [135]). (**B**) Schematic image showing restriction enzyme-responsive DNA hydrogel utilized for cell capture and release (adapted from Ref. [136]).

Restriction enzyme-responsive DNA hydrogels can be prepared by introducing specific sequences that are recognized and cleaved by restriction enzymes into the linker (Figure 9F). Restriction enzymes (or restriction endonucleases) are enzymes that recognize and cut specific DNA sequences, and are involved in the mechanism that protects bacteria from external genetic factors [134]. Restriction enzymes are classified into several types, and depending on the type, the recognition site, and the cleavage site may be a certain distance away. Restriction enzymes are typically recognizing specific DNA sequences of four to eight base pairs, catalyzing cleavage to produce either blunt or sticky ends. These restriction enzyme cleavages induce irreversible sol–gel conformational transitions, similarly to DNAzymes [23,48]. Xing et al. demonstrated that DNA hydrogels incorporating recognition sites for the restriction enzymes BamHI and EcoRI as linkers could be selectively degraded upon treatment with the corresponding restriction enzymes [23]. Lyu et al. achieved controlled drug release by utilizing linkers containing the recognition site for EcoRI [130]. Wang et al. further advanced this approach by employing restriction enzymes engineered to be activated through a cascade reaction in an inflammatory environment, thereby achieving controlled drug release [48]. Li et al. reported a more advanced restriction enzyme-based smart drug release system. They prepared a DNA hydrogel capable of combined gene therapy by simultaneously delivering Cas9/sgRNA RNP and DNAzyme using a pH-activated restriction enzyme (Figure 14A) [135]. This DNA hydrogel was prepared by RCA based on template DNA containing the sgRNA recognition sequence, DNAzyme, and the cleavage site of restriction enzyme, HhaI. By co-loading Mn^2+^ ion for DNAzyme activation and HhaI, which is activated in an acidic environment, a proton-activatable DNA-based nanosystem (H-DNC) was constructed. After being internalized by cancer cells, the HhaI enzyme is activated in the acidic environment, triggering the degradation of the DNA hydrogel and the release of both Cas9/sgRNA and DNAzyme. These two systems synergistically regulate gene expression, leading to potent anticancer effects. Designing it to be recognized by a specific restriction enzyme in this way can reduce nonspecific reactions, but requires a process for loading the restriction enzyme in an inactive state. Restriction enzyme-responsive DNA hydrogel can also be used to capture and separate cells. Jin et al. developed a single-cell capture-and-release platform utilizing a restriction enzyme-responsive DNA hydrogel cover (Figure 14B) [136]. They implemented a system that protects cells by utilizing DNA hydrogels formed by cross-linking of YDNA and linker DNA containing restriction enzyme cleavage sites, and then dissolves the hydrogel at the desired time by treating it with a restriction enzyme to release the cells.

## 5. Conclusions

DNA hydrogels are highly biocompatible materials and can be easily combined with a variety of other materials such as synthetic polymers and inorganic nanomaterials. Their mechanical stiffness can be precisely tuned by selecting different DNA building blocks and network cross-linking methods. Moreover, they offer specific molecular recognition capabilities and can incorporate functional linkers to respond reversibly and dynamically to various external stimuli. These unique structural and functional properties make DNA hydrogels highly promising materials for a wide range of applications in various fields. In this paper, we discussed various designs and working mechanisms of stimuli-responsive DNA linkers introduced into pure DNA hydrogel and hybrid DNA hydrogel systems and demonstrated their diverse biomedical applications, including controlled drug delivery, target analyte separation, protection and detection, and cell-free gene expression. In particular, DNA hydrogels integrated with stimuli-responsive DNA linkers can respond to a wide range of physical, chemical, and biological signals, such as pH, temperature, restriction enzymes, metal ions, small organic molecules, nucleic acid strands, proteins, target cells, and light. Furthermore, by combining them with other functional materials or incorporating multiple stimulus-responsive DNA linkers, these systems can be precisely programmed to operate under complex and diverse external stimuli, enabling the development of advanced smart systems. Additionally, DNA hydrogels are capable of carrying a wide variety of therapeutic payloads from conventional chemotherapeutics to nucleic acid therapeutics, positioning them as key materials for enabling personalized treatments and advancing precision medicine.

Despite numerous studies supporting the utility of DNA hydrogels, challenges such as low mechanical strength, limited physiological stability, poor cell permeability, and high production costs remain. Various approaches have been explored to address these issues, including the incorporation of synthetic polymers or inorganic nanomaterials, as well as chemical modification of the DNA itself. However, these strategies may still pose concerns due to the potential toxicity in vivo, accumulation-induced toxicity, immune responses, and uncertainties regarding long-term degradation profiles. Moreover, the complex design requirements for precision systems, along with the intricate processes and high costs associated with DNA synthesis, remain significant barriers to commercialization. In addition, in vivo studies on DNA hydrogels are still very limited, which represents a major obstacle to the practical clinical translation of their broad biomedical applications, including drug delivery, tissue engineering, and immunotherapy. However, ongoing research at the laboratory level continues to advance the field, with new systems being developed and validated at both the cellular and in vivo levels. These efforts highlight the strong potential of DNA hydrogels as promising materials for future biomedical innovations. Future research is expected to focus on overcoming these limitations through the development of biocompatible and biodegradable hybrid DNA hydrogels, the establishment of cost-effective large-scale DNA synthesis technologies, and the construction of sophisticated animal models to evaluate the long-term in vivo stability, immunological safety, and functionality of DNA-based materials. The direction of technological advancement is clear. Miniaturized, device-free detection systems suitable for point-of-care diagnostics remain one of the main goals. In addition, the separation, protection, and selective release of targets for in vitro analysis are crucial for precise disease diagnostics. Targeted delivery to specific cells and controlled drug release also continue to be essential research challenges aimed at reducing side effects of treatments. Furthermore, cell-based protein production and in vitro transcription (IVT) processes for mRNA synthesis still demand more efficient and automated cell-free gene expression systems, given their high consumption of time, resources, and infrastructure. Considering previous studies, DNA hydrogels are thought to have strong potential to develop into a versatile platform technology that can meet these biomedical needs when hybridized with various materials. As ongoing research continues to address the remaining challenges, DNA hydrogels are expected to emerge as a key research area for the development of next-generation biomaterials.

## Data Availability

Not applicable.
